# Pulmonary tuberculosis masquerading as a mass cancer lesion: A rare case report and review of literature

**DOI:** 10.1002/ccr3.9155

**Published:** 2024-07-03

**Authors:** Gohar Fatima, Zafar Ahmed, Ayesha Akhtar, Syed Tabish Rehman, Sajjad Ali, Mahfuza Anan

**Affiliations:** ^1^ Department of Chest Medicine and Critical Care Liaquat National Hospital and Medical College Karachi Pakistan; ^2^ Department of Chest Medicine and Critical Care Dr. Ziauddin University Hospital Karachi Pakistan; ^3^ Department of Thoracic Surgery Liaquat National Hospital and Medical College Karachi Pakistan; ^4^ Department of Medicine Dr. Ziauddin University Hospital Karachi Pakistan; ^5^ Department of Medicine Bangladesh Medical College Dhaka Bangladesh

**Keywords:** biopsy, mass lesion, pulmonary tuberculosis, radiology

## Abstract

**Key Clinical Message:**

Despite being generally treatable and preventative, pulmonary tuberculosis (PTB) is one of the most common infectious agents that cause death. Misdiagnosis of TB frequently leads to unwarranted diagnostic procedures and postpones the start of treatment.

**Abstract:**

Pulmonary tuberculosis (PTB) can present with various unusual radiological and clinical characteristics. Misdiagnosis of TB frequently leads to unwarranted diagnostic procedures and postpones the start of treatment. Here, we describe a 50‐year‐old man who presented with a cancerous‐type lesion on radiological findings and atypical symptoms that led to an initial diagnosis of lung cancer. However, histopathology and biopsy of the lung lesion revealed chronic granulomatous inflammation with caseous necrosis, confirming PTB as the true cause, with no further indications of malignancy.

## 1 INTRODUCTION

In 2022, 1.3 million people globally succumbed to death from pulmonary tuberculosis (PTB). PTB is the second most common infectious agent that causes death, behind HIV/AIDS, and the thirteenth most common cause of death overall.^1^ As a general rule, PTB can be diagnosed by looking for acid‐fast bacilli (AFB) in stained sputum.^2^, ^3^ However, AFB smear results could be negative or ambiguous, and it can be challenging to diagnose a patient early if the condition is not detected by a physician or through radiological means.

When it comes to lowering the morbidity and mortality linked to PTB, early diagnosis is equally important as treatment. For the diagnosis of TB, transthoracic biopsy has been shown to be useful.^3^ Moreover, TB often presents atypical radiologic findings^4^; among the rare radiographic indicators of PTB reported are mass lesions resembling neoplasms.^5^, ^6^

This study aims to present a unique case study of a male patient, aged 50, who had PTB that appeared as a mass lesion on radiological examination, delaying the treatment of PTB.

## 2 CASE PRESENTATION

### 2.1 History and examination

A 50‐year‐old male with a known case of Diabetes Mellitus for 3 years (compliant on oral hypoglycemic drugs) presents with a 14‐day history of non‐productive cough, along with high‐grade fever and right‐sided sharp pleuritic chest pain localized to the lower chest. The fever was sudden in onset, progressing from low‐grade to high‐grade over time, continuous without rigors or chills, and mildly relieved by medication temporarily. The patient had previously taken Oral Cefixime and Clarithromycin for 4 days, but no signs of improvement were noticed.

On general physical examination, the patient had a 103 F temperature and maintained oxygen saturation (SO_2_) at room air. On respiratory examination, a dull percussion note was noted at the fourth to fifth intercostal space, with bronchial breathing and increased vocal resonance at the site.

### 2.2 Laboratory and radiological findings

Laboratory investigation revealed a total leucocyte count (TLC) of 21,000/μL, C‐reactive protein (CRP) of 113 mg/L, and erythrocyte sedimentation rate (ESR) of 59 mm/h; the rest of the lab workup was unremarkable, with normal renal functions, electrolytes, and liver function.

**FIGURE 1:**
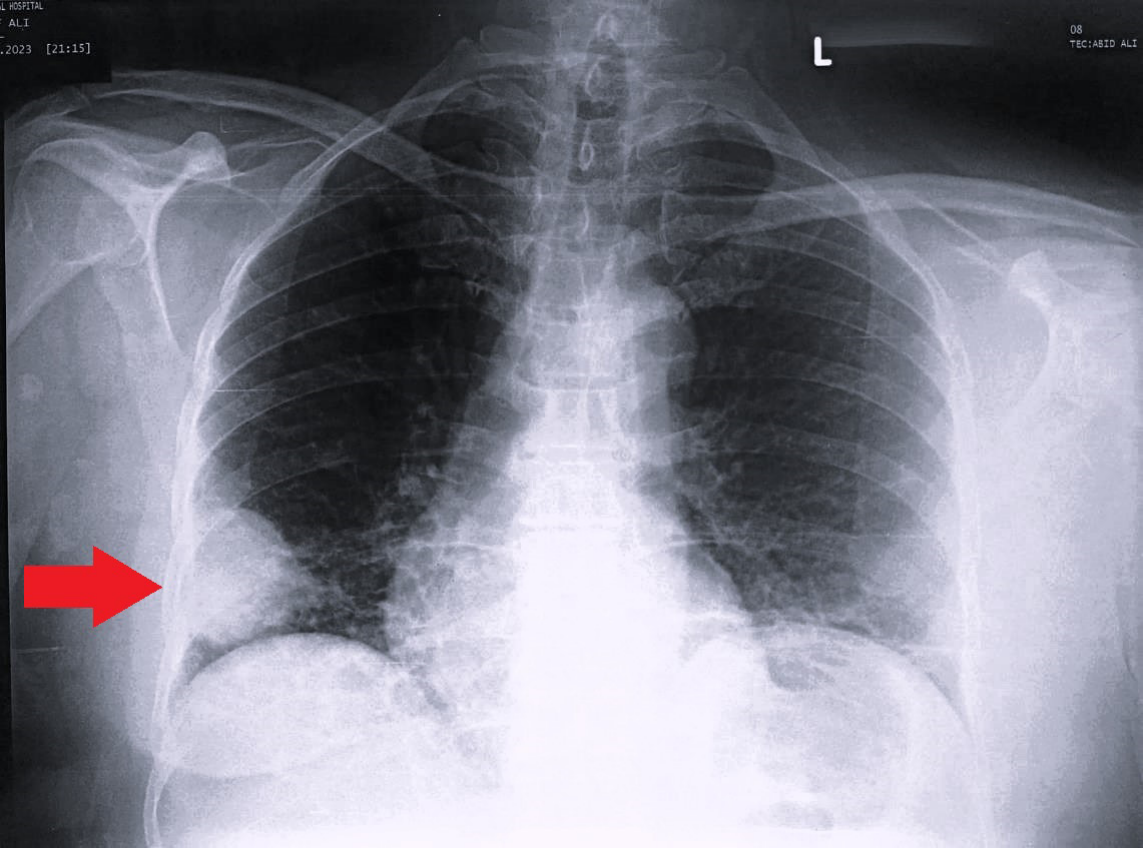
A posterior–anterior (PA) chest x‐ray showing a right lower lobe mass lesion.

A chest x‐ray (CXR) posterior–anterior (PA) view was performed, which showed a well‐circumscribed homogenous opacification in the right mid‐lower zone, giving an appearance likely of a mass lesion (Figure 1).

The patient was hospitalized and initiated on Intravenous Ceftriaxone 2 g once a day. Additionally, the administration of Clarithromycin was continued at the exact dosage, keeping pneumonia as an initial differential diagnosis in mind.

**FIGURE 2:**
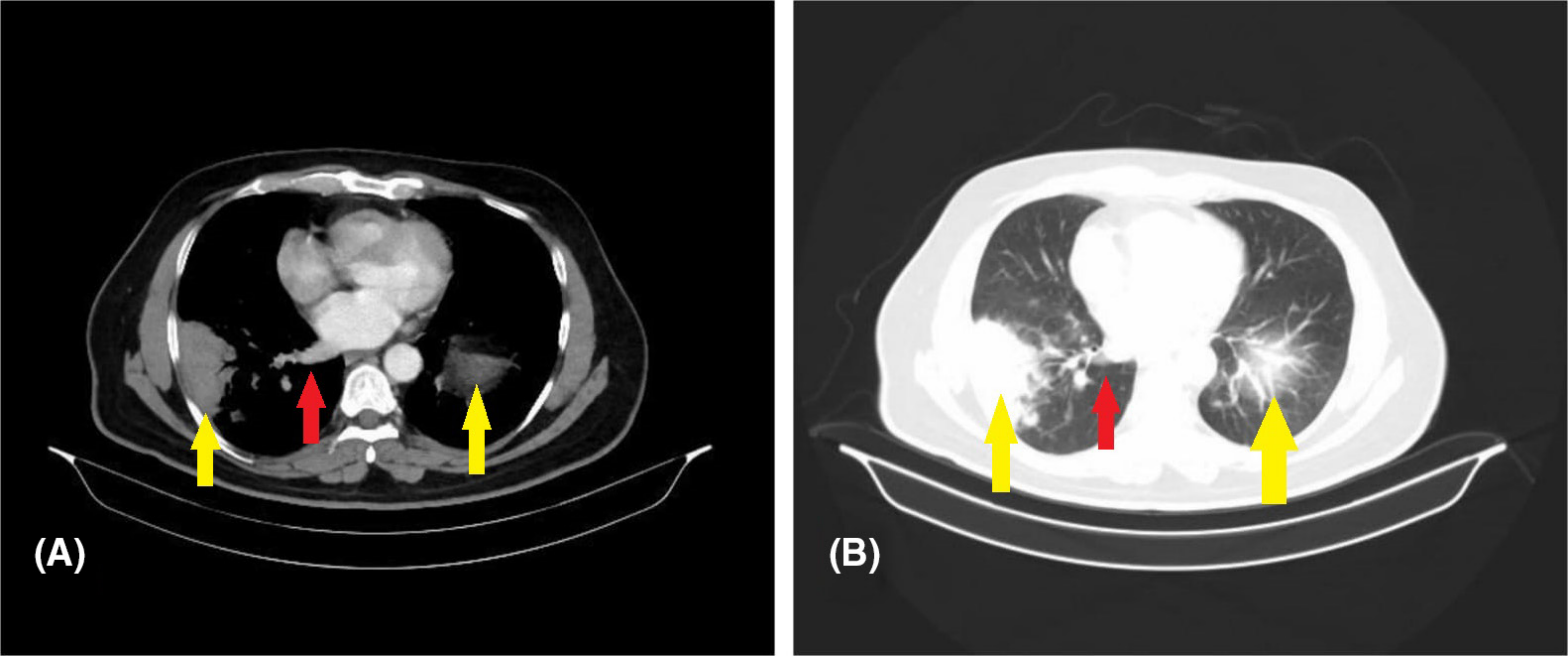
(A) CT scan of the chest with contrast and (B) CT scan of the chest without contrast, showing soft tissue density in the lower base of the right lung (yellow arrow), accompanied by an enlargement of the right hilar lymph node (red arrow).

A chest computed tomography (CT) scan with contrast revealed the presence of numerous pulmonary nodules dispersed throughout the right lung (Figure 2). Additionally, patchy inflammatory alterations were observed at the base of the left lung. A region of soft tissue density was identified in the lower base of the right lung, accompanied by an enlargement of the right hilar lymph node measuring approximately (24 × 12 mm) in diameter. Furthermore, multiple prominent lymph nodes were detected in the right axilla, with the largest measuring about (23 × 9) mm in diameter.

### 2.3 In‐hospital treatment and clinical diagnosis

**FIGURE 3:**
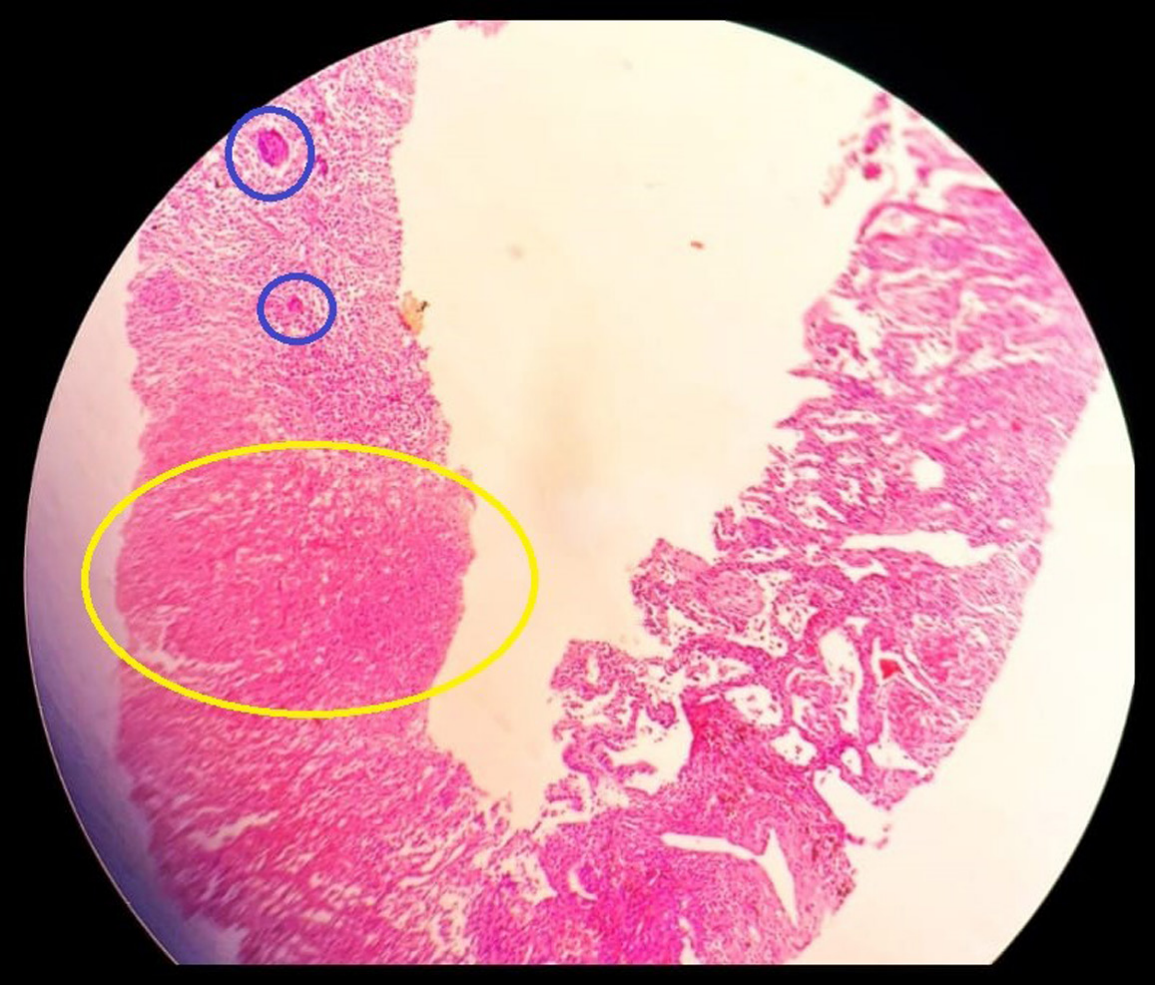
CT‐guided lung biopsy revealing the presence of chronic granulomatous inflammation characterized by Langerhans‐type multinucleated giant cells (blue circle) and widespread areas of caseous necrosis (yellow circle).

The patient consistently experienced episodes of elevated body temperature, characterized by high‐grade fevers that were mildly alleviated with the administration of paracetamol. Furthermore, since the patient had no expectorant, the AFB smear test could be conducted. Therefore, the patient underwent a lung biopsy procedure with CT guidance on the subsequent day to rule out if the lesion was either a cancerous origin or a TB origin. The lung biopsy report (Figure 3) revealed the presence of chronic granulomatous inflammation characterized by Langerhans‐type multinucleated giant cells and widespread areas of caseous necrosis.

Given the history, radiological picture, histopathology findings, and keeping in view the high prevalence of tuberculosis in the area, the patient was diagnosed with PTB.

### 2.4 Treatment and outcome

**FIGURE 4:**
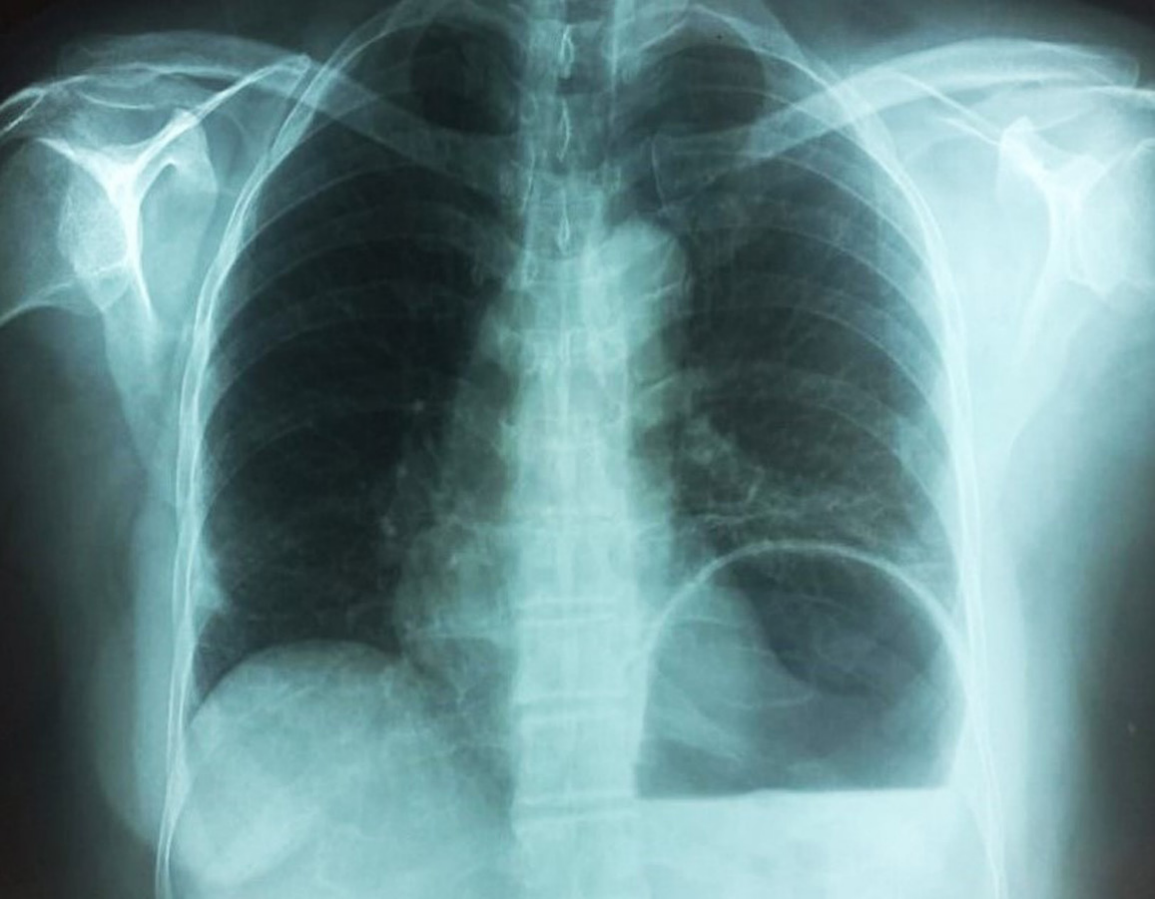
Chest x‐ray of the patient on the 4th week of post‐treatment showing improvement.

The patient was shifted to an isolated room and started on an anti‐tuberculosis treatment regime, including rifampicin, isoniazid, ethambutol, and pyrazinamide in weight‐adjusted dosages. The patient was kept under observation for a week until the symptoms resolved. After 4 weeks, the patient was observed in a pulmonology clinic, during which a follow‐up chest x‐ray was conducted (Figure 4 **)**. The x‐ray results indicated a significant improvement in the consolidation observed compared to the prior x‐ray.

## 3 DISCUSSION

PTB has been mistakenly diagnosed as lung cancer, leading to misdiagnosis. PTB is also characterized by chest imaging abnormalities suggestive of lung malignancy, such as irregularly shaped consolidations with cavities with thick walls on CT imaging. It is, therefore, impossible to offer a radiological foundation for distinguishing the two entities. Microbiological and pathological testing for correlation is needed to confirm the diagnosis.

According to a study by Prytz et al. 91 individuals who had thoracotomy with an assumed diagnosis of lung cancer were later found to have pulmonary tuberculosis.^7^ Moreover, a study by Shan Lang et al. reported 22 patients with PTB mimicking lung cancer on radiological chest CT findings.^8^ A recent study by Tan, K T et al. in Malaysia, showcased a similar case report of a 67‐year‐old male TB patient presenting as a mass lesion on radiological imaging.^9^

Over a period of 3 years, Rolston et al. conducted a retrospective review on patients who were first thought to have lung cancer but ultimately had pulmonary infections. Fungal infections made up 46% of these illnesses, followed by mycobacteria (27%), bacteria (22%), and parasitic infections (5%).^10^

Kassam, Nadeem M et al. reported another case that highlighted an uncommon presentation of tuberculosis, characterized by extensive atelectasis resulting from an endobronchial polypoid lesion.^11^ These findings are consistent with the findings of our case report. Based on the patient's medical history, radiological imaging, and histopathological examination results, initiating anti‐tuberculous therapy was deemed appropriate. This decision was made considering the high incidence of TB in the geographical area.

In addition, a study by Paul Chhar Bun showcased a 26‐year‐old female patient presented with a diagnosis of endobronchial tuberculosis.^12^ The patient had symptoms of a lung mass along with a lesion on the spine that resembled metastasis. The CT scan of the chest revealed the presence of a mass in the left lower lobe, which was accompanied by the destruction of the vertebral body of T11. The histopathological findings of the lesion indicated the presence of an inflammatory response in the granuloma tissue, which is indicative of a potential tuberculous infection.^12^

Accurately differentiating between PTB and lung cancer is still a diagnostic challenge in the clinic due to the nonspecific clinical signs and radiologic markers of PTB.^13^ PTB's typical symptoms are typically simple to diagnose because of well‐defined clinical traits and radiographic findings. Nonetheless, a number of PTB symptoms, such as hemoptysis, weight loss, and cough, are also frequently associated with lung cancer.^14^ However, PTB also exhibits characteristic radiographic signs of lung cancer, such as thick‐walled cavities.^13^ As a result, patients with PTB who mimic lung cancer are frequently misdiagnosed with lung cancer. The mistake could lead to ineffective surgeries and postponed PTB treatment, which would worsen the condition's severity and complications.

## 4 CONCLUSION

To sum up, individuals who have PTB that mimics lung cancer typically present with an aberrant chest CT finding, which is quite comparable to lung cancer. Clinicians should have a high index of suspicion for PTB in TB‐endemic regions where lung cancer and TB can be mistaken for one another on clinical and radiological grounds. The distinction between TB and lung cancer should not be made only based on radiographic imaging. Microbiological and histological testing are essential to confirm the diagnosis of TB in patients with atypical manifestations.

## AUTHOR CONTRIBUTIONS

**Gohar Fatima:** Conceptualization; formal analysis; investigation; methodology; project administration; visualization; writing – original draft. **Zafar Ahmed:** Conceptualization; data curation; investigation; methodology; validation; writing – original draft. **Ayesha Akhtar:** Data curation; methodology; resources; validation; writing – original draft. **Syed Tabish Rehman:** Conceptualization; formal analysis; investigation; project administration; software; visualization; writing – original draft. **Sajjad Ali:** Investigation; project administration; supervision; validation; writing – original draft; writing – review and editing. **Mahfuza Anan:** Conceptualization; methodology; project administration; writing – review and editing.

## FUNDING INFORMATION

None.

## CONFLICT OF INTEREST STATEMENT

None of the authors have any conflict of interest.

## ETHICS STATEMENT

Our institution does not require ethical approval for reporting individual cases or case series.

## CONSENT

Written informed consent was obtained from the patient to publish this report in accordance with the journal's patient consent policy.

## Data Availability

The data that support the findings of this study are available on request from the corresponding author. The data are not publicly available due to privacy or ethical restrictions.
